# Investigation of Transport in OECTs with Electrochemical Strain Wave Microscopy

**DOI:** 10.1002/smll.202510345

**Published:** 2025-11-14

**Authors:** Filippo Bonafè, Junpeng Ji, Simone Fabiano, Beatrice Fraboni, Tobias Cramer

**Affiliations:** ^1^ Department of Physics and Astronomy University of Bologna Viale Berti Pichat 6/2 Bologna 40127 Italy; ^2^ Laboratory of Organic Electronics Department of Science and Technology Linköping University Norrköping SE‐601 74 Sweden

**Keywords:** electrochemical strain waves, modulated electrochemical atomic force microscopy, organic electrochemical transistors, organic mixed ionic electronic conductors

## Abstract

Understanding microscopic mixed ionic‐electronic conduction in organic electrochemical transistor (OECTs) is crucial to advance bioelectronic, neuromorphic, and sensing applications. In this work, electrochemical strain wave (ESW) microscopy is introduced as a novel approach to probe transport processes in the OECT channel at microscopic length scales. ESWs are generated in mixed conductors by charge injection at the electrodes and propagate into the device channel through combined charge transport and swelling. With AFM experiments, the local amplitude and phase of ESWs are mapped in OECTs under different operating conditions. Results demonstrate that quantitative ESW acquisitions can determine carrier concentration‐dependent mobility effects in n‐type and p‐type devices, or reveal microstructural defects in the OECT channel, altering local charge transport. The findings provide a coherent framework to interpret the structure‐functionality relation in OECTs, mapping transport properties below the diffraction limit and without limitations by screening effects or electrochemical side reactions of the probe.

## Graphical abstract

Electrochemical strain waves (ESWs) are generated in mixed conductors by alternating charge injection at a contacting electrode. They propagate into the device channel through combined charge transport and swelling. AFM experiments map the local amplitude and phase of ESWs in organic electrochemical transistors (OECTs), providing quantitative insight on microscopic transport properties.

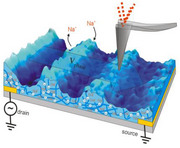

## 1 Introduction

Organic electrochemical transistors (OECTs) offer great potential for bioelectronics, neuromorphic computing,^[^ ^1^, ^2^, ^3^, ^4^ ^]^ electrochemical sensing,^[^ ^5^, ^6^ ^]^ and logic circuit integration^[^ ^7^, ^8^ ^]^ due to device biocompatibility,^[^ ^9^ ^]^ integration of ionic and electronic signals,^[^ ^10^, ^11^ ^]^ and facile device processing with additive methods.^[^ ^12^, ^13^ ^]^ OECTs make effective use of ion injection from an electrolyte in contact with the organic mixed ionic‐electronic conductor (OMIEC) channel to modulate its electronic conductivity.^[^ ^14^ ^]^ The effect is controlled by the electrochemical gate potential that sets the doping level (redox state) of the OMIEC layer.^[^ ^15^ ^]^ Events that alter the electrochemical potential and impact on the ionic distribution at the channel–electrolyte interface cause a large variation in the number of electronic carriers flowing through the channel from source to drain electrode, hence leading to an amplification effect.^[^ ^16^, ^17^ ^]^ Recent advancements in device architectures^[^ ^18^, ^19^, ^20^ ^]^ and the formulation of novel OMIEC materials^[^ ^21^, ^22^ ^]^ have led to significant improvements in device performances, but further progress in the field is limited by some open questions on the fundamental processes determining the OECT operation. Charge accumulation and transport in the OMIEC channel involve ionic and electronic charge carriers, which highly interact in the entire film bulk.^[^ ^23^ ^]^ The mixed conductivity renders the experimental characterization of individual ionic or electronic transport difficult,^[^ ^24^ ^]^ and it is still unclear how these processes impact on the local screening of electric fields^[^ ^25^ ^]^ or introduce contact resistances in the OECT.^[^ ^26^, ^27^ ^]^ Further studies are required to understand how transport is affected by variations in the OMIEC morphology induced by material processing^[^ ^28^ ^]^ and synthetic microstructure^[^ ^21^ ^]^ or how local defects in the polymeric matrix determine the overall device properties.^[^ ^29^ ^]^ Progress on all fronts requires the introduction of novel microscopic techniques to inquire on local charge transport in OECTs under operating conditions.

Microscopic mixed conduction processes in OECTs are typically studied using optical methods such as the moving redox front experiment.^[^ ^30^ ^]^ These monitor real‐time changes in the color of the active OMIEC channel caused by gradual electrochemical (de)doping driven by lateral ion injection.^[^ ^31^ ^]^ Recent moving front approaches based on operando visible microscopy were used to study transport limitations in OECTs and revealed slow electronic transport effects impacting on the functionality of accumulation‐mode devices.^[^ ^15^, ^32^ ^]^ Other relevant characterization methods involve Raman mapping of polaronic vibrations across the channel to visualize the carrier distribution in operating devices,^[^ ^33^ ^]^ or in situ X‐ray diffraction and NMR to reveal, respectively, microstructure variations^[^ ^34^ ^]^ and changes in ion coordination^[^ ^35^ ^]^ during electrochemical gating. Also, scanning electrochemical microscopy (SECM) experiments were successfully performed to probe the local electrochemical potential in the OECT channel, obtaining information on the electronic carrier mobility.^[^ ^36^ ^]^ However, these methods usually require millimeter‐sized device channels and can only reveal transport processes occurring on the timescale of seconds. Due to the diffraction limit of the optical probes, they lack of sufficient spatial resolution to monitor how OECT transport is affected by the OMIEC channel properties at the micro/nanoscale.

To address these challenges, scanning‐probe microscopy techniques offer a unique capability to characterize devices during operation and establish structure‐function relationships characteristics over a broad range of scales (from nano‐ to micrometer device scales).^[^ ^37^ ^]^ In this contest, Kelvin probe force microscopy (KPFM) experiments are typically used to determine the local electrical potential distribution^[^ ^38^ ^]^ and charge trapping dynamics^[^ ^39^ ^]^ in organic field‐effect transistors (OFETs).^[^ ^37^ ^]^ However, efficient KPFM measurements in electrolyte‐gated devices have not yet been demonstrated due to bias‐induced screening processes and electrochemical side reactions occurring at the interface with the electrolyte.^[^ ^40^ ^]^ Alternatively, in‐liquid scanning dielectric microscopy (SDM) has been recently introduced for the measurements of local conductivity and interfacial capacitance of electrolyte‐gated organic field‐effect transistors (EGOFETs) during operation.^[^ ^29^ ^]^ Nevertheless, SDM experiments require the formation of a 2D charge transport layer in the transistor channel at the semiconductor/electrolyte interface^[^ ^41^ ^]^ and thus cannot be performed on OMIEC materials, which are permeable to ions and exhibit electronic conduction in the entire film bulk. On the other hand, electrochemical strain microscopy (ESM) can directly probe local ion uptake and consequent electrochemical oxidation of the organic semiconductor by measuring sub‐nanometric volumetric expansion of the OECT channel.^[^ ^42^ ^]^ However, in SDM and ESM experiments, the conductive AFM tip is used to generate the local electric field. Consequently, the field distribution is not linear, and electrochemical reactions cause additional polarization effects on the metallic AFM tip. Accordingly, it is inherently difficult to achieve a quantitative analysis of these measurements. A different situation is exploited in electrochemically controlled AFM (EC‐AFM) experiments, as here the potential (and the electric field) is controlled by reference and counter electrode operated with a potentiostat, while the AFM probe is not electrically contacted, but acts solely as a non‐conductive nanomechanical transducer to measure changes in the sample's surface morphology. As a consequence, EC‐AFM experiments are not affected by ionic screening effects or electrochemical reactions at the probe and allow for monitoring charge exchange processes occurring in the entire OMIEC film bulk.^[^ ^43^ ^]^ In recently introduced modulated electrochemical atomic force microscopy (mEC‐AFM), a small AC signal is added by the potentiostat, allowing to obtain quantitative information on local ionic transport into the OMIEC material and related swelling effects.^[^ ^44^, ^45^ ^]^ However, mEC‐AFM protocols probing the interplay between local ionic transport and OECT operation have still to be established.

In this work, we introduce electrochemical strain wave (ESW) microscopy as a novel method relying on atomic force microscopy to map charge transport in OECTs. We demonstrate how the propagation of electrical signals in the OECT generates dynamic volume changes in the channel that propagate as an electrochemical strain wave. Through AFM experiments under electrochemical control, we scan the local amplitude and phase of strain waves generated at the electrical contacts of OECTs in normal operating conditions. We develop a quantitative model for experimental data interpretation, allowing us to determine carrier concentration‐dependent mobility effects in n‐type and p‐type OECTs. By comparing microstructural images of the sample topography with quantitative ESW acquisitions, we demonstrate how microscopic defects in the channel thin film affect the local distribution of charge carriers and electrical potential in the OECT. Our findings provide a coherent framework to interpret the (nano)structure‐functionality relation in operating OECT devices, also highlighting intrinsic differences in charge transport and energy dissipation processes between p‐type and n‐type transistors.

## 2 Results

### 2.1 Electrochemical Strain Waves in OECTs

**Figure 1:**
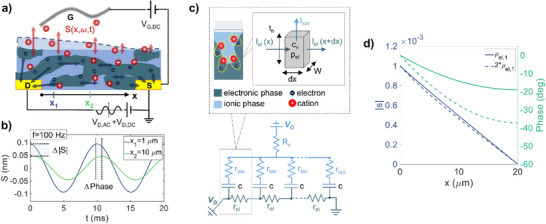
Electrochemical strain waves in OECTs. a) An AC voltage applied to the drain electrode causes local variations in ionic and electronic carrier density. These lead to the local swelling *S(x,ω)* of the OMIEC material and generate an electrochemical strain wave propagating into the OECT channel. b) Swelling oscillations simulated at two different points on the channel surface show the effect of attenuation and phase delay of the strain wave due to transport through the dispersive ionic medium. c) Finite volume element discretization of an n‐type OMIEC thin film. Electronic and ionic carriers are represented in spatially separated ionic and electronic phases constituting the material nanostructure. The OECT channel is modeled for small‐signal analysis by an electrical transmission line. d) Impact of materials transport properties on strain waves: amplitude and phase of the electrochemical strain wave (defined as the swelling per unit of thickness *s*) as a function of channel position *x* for two different channel resistivities (*ρ_el_* = 1 Ωcm (continuous line) and *ρ_el_* = 2 Ωcm (dashed line)).

The central hypothesis of our work is that ESWs resulting from mixed‐conduction processes in the channel provide information on charge transport in OECTs. **Figure** **1**a introduces the concept of electrochemical strain waves in a schematic cross‐section of an n‐type OECT. In this configuration, a small voltage modulation *V_D,AC_* =  *V_in_e* ^*j*ω*t*^ is applied to the drain electrode and added to the DC drain voltage at time *t* = 0 and position *x* = 0. The resulting AC signal propagates along the OMIEC channel (with length *L*, width *W*, and thickness *t_h_*) and reaches the OECT source, which is grounded. Source and drain gold contacts act as selective contacts for electronic carriers, while the inert substrate and the electrolyte bath constitute a selective contact for ionic carriers. The propagating AC potential wave alters the local charge distribution through the injection/ejection of mobile ions, causing dynamic volume changes in the channel that propagate as an electrochemical strain wave along with the electrical signal. As a result, swelling oscillations *S*(*x,ω*) are present on the surface of the channel depending on the position *x* and on the angular frequency *ω* of the AC modulation. As an example, Figure 1b compares two swelling signals, *S_1_* and *S_2_* at two different points separated by 10 µm along the horizontal profile of the channel (with length *L* = 20 µm). The finite velocity of the wave causes the phase delay between *S_1_* and *S_2_*. The dispersion of energy into the surrounding ionic environment leads to the attenuation of the wave's amplitude with increasing distance from the injection point.

To derive a mathematical description of the electrochemical strain wave propagation, we consider the ionic and electronic transport processes in mixed conductors generally modeled using drift diffusion approaches.^[^ ^46^ ^]^ In such approaches, the mixed conductor^[^ ^25^ ^]^ is described by infinitesimal volume elements, as shown for an n‐type mixed conductor in Figure 1c. Each volume element is characterized by the electronic and ionic resistivity ρ_*el*_ and ρ_*ion*_,^[^ ^47^ ^]^ and a volumetric capacitance *c_v_* describing how changes in the chemical potential impact on the accumulation of ionic and electronic carriers.^[^ ^24^ ^]^ Recent works studying signal propagation in electrolyte‐gated OMIEC channels demonstrate that a material consisting of mixed conductor volume elements can be accurately represented for small‐signal analysis by an equivalent circuit model corresponding to an electrical transmission line.^[^ ^48^, ^49^ ^]^ Such a description can be extended for OECTs, considering the electrical circuit in Figure 1c. Electrical transport in the horizontal direction of the device channel occurs along the electronic carrier path described by the electronic resistance per unit length, *r_el_* (measured in Ω m^−1^). The electronic path is coupled through the volumetric capacitance to the ionic transport path in the *y*‐direction characterized by the ionic resistance per unit thickness *r_ion_*. *R_s_* finally indicates the ionic resistance of the surrounding electrolyte where the gate voltage is applied. The propagation of an electrical potential wave along the circuit in Figure 1c can be described using the transmission line theory.^[^ ^49^, ^50^ ^]^ For small signal analysis, we demonstrate in Section T1 (Supporting Information) that the local electrochemical strain in the OECT channel *s*(*x*, *t*) = *S*(*x*, *t*)/*t_h_* (defined as the swelling per unit thickness of the OMIEC film) is directly proportional to the traveling AC voltage signal and satisfies the cable equation^[^ ^50^ ^]^

(1) $$\frac{d^{2}s\left(x,t\right)}{dx^{2}}=\gamma^{2}s\left(x,t\right)$$

where the propagation constant *γ* is defined as the ratio between the horizontal impedance *r_el_* and the vertical (or characteristic) impedance *z_0_* of the line:^[^ ^51^ ^]^

(2) $$\gamma =\sqrt{\frac{r_{el}}{z_{0}}}$$

A general solution for Equation (1) is given by

(3) $$s\left(x,t\right)=s\left(x\right)e^{j\omega t}$$

corresponding to a wave equation where the spatial term *s(x)* is determined by the chosen set of boundary conditions (see Section T1, Supporting Information). Based on the scheme in Figure 1a, we can impose: i) the applied AC potential generates a strain *s(x = 0,t = 0) = s_0_* on the drain electrode, and ii) the source electrode is grounded and acts as a node *s(x = L, t = 0)= 0* for the strain wave, giving

(4) $$s\left(x\right)=\frac{s_{0}}{\sinh\left(\gamma L\right)}\sinh\left[\gamma \left(L-x\right)\right]$$

An animation showing the spatiotemporal propagation of the ESW resulting from Equations (3) and (4) in the OECT channel is provided as multimedia material. An equivalent description of the process in the frequency domain can be obtained by considering the phasor $\bar{s}\left(x\right)=\left|s(x)\right|e^{j\phi (x)}$ associated with the local film strain. Figure 1d reports a simulated example for the spatial maps of amplitude and phase of $\bar{s}\left(x\right)$ when an AC voltage with frequency *f* = 100 Hz is applied to the drain electrode. Results plotted for different channel resistivities *ρ_el_* reveal that an increase in *ρ_el_* by a factor of two (dashed lines) produces only a small variation of the local strain amplitude but causes a significant increase in the phase delay of the signal along the channel. This observation highlights that the strain wave phase is particularly sensitive to variations in transport properties in the OECT device.

### 2.2 Experimental Observation of Electrochemical Strain Waves in OECT Channels

**Figure 2:**
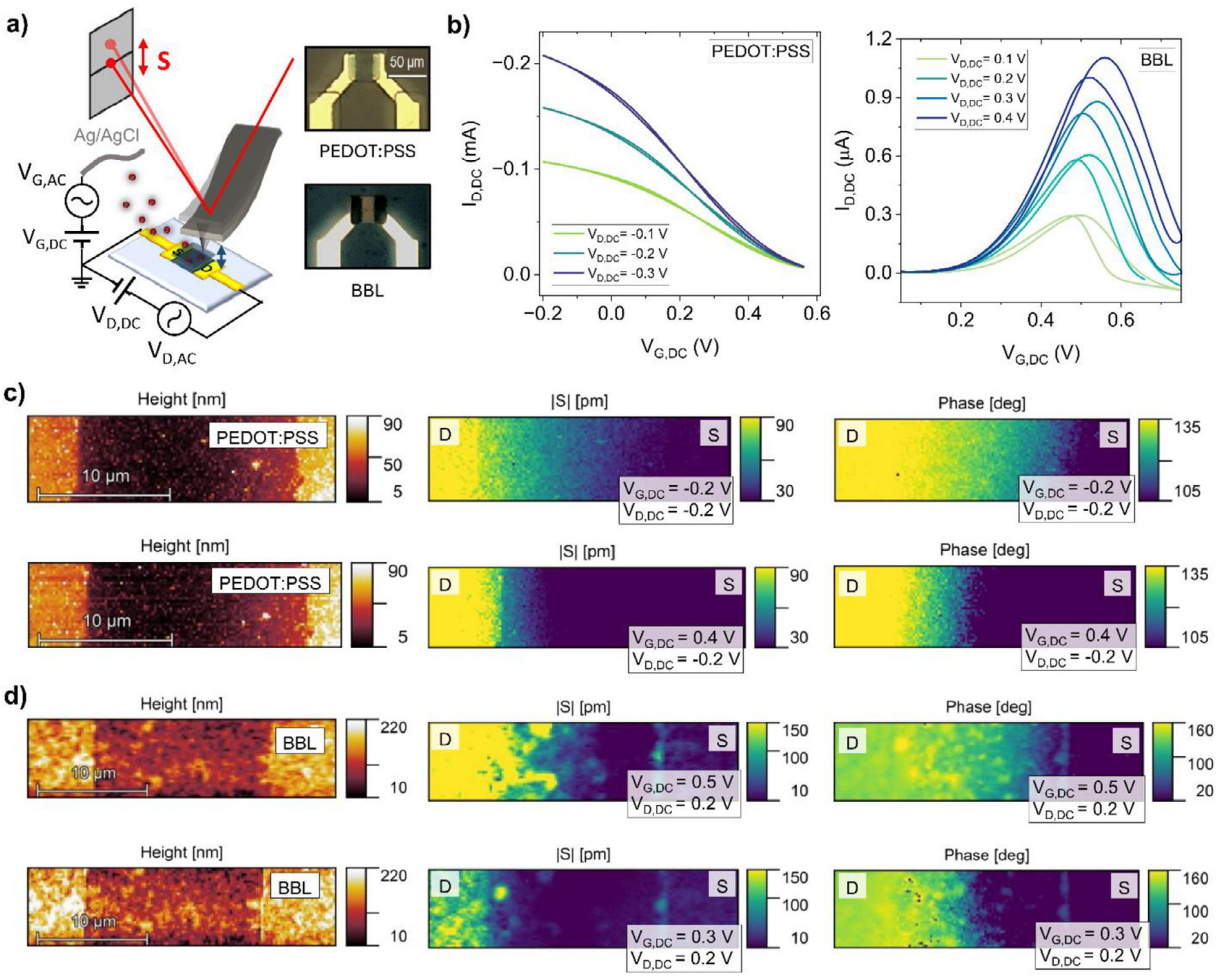
Experimental observation of electrochemical strain waves (ESWs) in OECT channels. a) Schematic of the experimental apparatus for mEC‐AFM measurements of electrochemical strain waves propagating in OECT channels. Optical microscopy images of OECT devices based on PEDOT:PSS and BBL are reported as an inset. b) DC transfer curves of made with PEDOT:PSS or BBL as channel material. c) mEC‐AFM maps of surface topography, ESW amplitude *S*, and ESW phase in PEDOT:PSS OECTs. The AC modulation generating the ESW is injected from the drain electrode positioned at the left of the AFM images (drain and source electrodes are labeled in the top part of the images). The upper and lower row of maps compare a more conducting PEDOT: PSS channel (*V_G,DC_* = ‐0.2V) to a less conducting channel (*V_G,DC_* = 0.4). d) Same as (c) but for the BBL based OECT. The two rows compare the conducting BBL channel (*V_G,DC_* = 0.5 V) to the less conducting channel (*V_G,DC_* = 0.3 V). For both materials, a more rapid attenuation of the ESW amplitude and larger phase loss (i, j) is measured when the electronic conductivity of the OMIEC channel is decreased.

The model for electrochemical strain waves introduced in Figure 1 predicts that the local amplitude and phase of the wave can inform on the electronic and ionic transport properties within the OECT channel. To test this hypothesis, we use the modulated electrochemical atomic force microscopy (mEC‐AFM) technique in‐operando on OECT channels. A schematic of the setup used for the experiments is reported in **Figure** **2**a. Measurements were performed on both p‐type and n‐type OECTs based on poly(3,4‐ethylenedioxythiophene):polystyrene sulfonate (PEDOT: PSS)^[^ ^27^ ^]^ and poly(benzimidazobenzophenanthroline) BBL^[^ ^52^ ^]^, respectively. Optical micrographs of samples showing the microstructured OECT channels (with width *W* = 30 µm and length *L* = 20 µm) resulting from the microfabrication process described in the Methods section are reported as an inset. Devices are immersed in a 0.1 m PBS solution, using an Ag/AgCl wire as the gate electrode. The OECT operation is enabled by applying DC voltages *V_D,DC,_* and *V_G,DC_* to the drain and gate electrodes. The resulting OECT transfer plots are reported in Figure 2b,c, and reproduce the typical features reported for such devices in the literature.^[^ ^53^, ^54^ ^]^ As PEDOT:PSS OECTs are based on an intrinsically doped organic semiconductor, they operate in depletion mode, while BBL n‐type transistors require positive gate voltages to accumulate electronic carriers in the channel.^[^ ^55^ ^]^ In addition, in BBL based OECTs, larger gate voltages (*V_G,DC_* >0.5 V) cause a rapid decay in DC drain current, giving rise to a maximum in the transfer characteristics termed as “antiambipolar” behavior.^[^ ^56^ ^]^

To gain evidence for the predicted ESWs in these two OECT devices, we apply an AC voltage signal to the drain electrode and perform mEC‐AFM imaging to map the channel region pixel per pixel with fast force‐distance spectroscopy measurements. While in contact with the surface, a lock‐in amplifier filters the cantilever deflection signal *D* containing the local oscillations of the material surface.^[^ ^44^, ^45^ ^]^ In this way, we obtain maps of the amplitude *S* and phase shift *ϕ_S_* of the OMIEC swelling with respect to the AC signal injected at the drain electrode. In addition to the AC signal (amplitude |*V_D,AC_* | = 100 mV, frequency *f* = 3.1 kHz and *f* = 1.7 kHz for PEDOT: PSS and BBL samples, respectively – see Methods for further details), we apply DC offset potentials to the gate and drain electrodes allowing us to acquire maps of the local electroswelling amplitude and phase at different OECT working points. No significant variations in the DC transfer and output characteristics were observed upon applying the AC modulation to the drain (see Section S7, Supporting Information), confirming that *V*_*D*,*AC*_ does not influence the steady‐state operation of the OECTs. mEC‐AFM images with 128x32 pixels covering an area of 30x7.5 µm of the OECT channels are reported in Figure 2c,d. The height maps illustrate the morphology of the transistor channels. The channel length (≈20 µm) is defined by the underlying gold source and drain electrodes (with a thickness of ≈50 nm) appearing at the right and left sides of the image, respectively. PEDOT: PSS samples (Figure 2c) exhibit a relatively uniform microstructure, while BBL channels (Figure 2d) show a rougher morphology composed of larger material grains. The related maps of ESW amplitude and phase were acquired at two different gate potentials to investigate how the doping level of the organic semiconductor influences ESW propagation. High doping levels and channel conductivity were obtained with operating conditions set at *V_G,DC_*  = −0.2 V, *V_D,DC_*  = −0.2 V for PEDOT:PSS devices and *V_G,DC_* = 0.5 V, *V_D,DC_* = 0.2 V for BBL OECTs, respectively. These voltage values were selected based on the corresponding transfer characteristics (Figure 2b), which show a maximum in the drain current under these bias conditions. For both p‐type (Figure 2c) and n‐type OECTs (Figure 2d), the electroswelling amplitude and phase assume a constant and maximum value in correspondence to the drain electrode (left side of the images), and then gradually drop along the transistor channel, reaching a minimum close to the source. At the same time, for lower channel conductivities (*V_G,DC_* = 0.4 V, *V_D,DC_* = −0.2 V for PEDOT:PSS OECTs and *V_G,DC_* = 0.3 V, *V_D,DC_* = 0.2 V for BBL OECTs – as indicated by the transfer curves in Figure 2b), the sample morphology remains unaltered, but the swelling amplitude front is mostly localized in the proximity of the drain electrode, and the phase rapidly decays along the horizontal direction *x*. Such results provide microscopic evidence of the mechanism described in Figure 1. When the OECT channel is highly doped, charge transport occurs mostly along the electronic conductor of the OMIEC material (represented by the electronic circuit in Figure 1b), and the AC potential responsible for the electrochemical strain drops linearly along the channel. Increasing the electronic resistivity of the organic semiconductor increases the amount of charge dissipated in the ionic circuit. As a consequence, ESWs propagating in the OMIEC layer slow down and experience a more rapid spatial attenuation: the phase shift is more pronounced and the amplitude of the local swelling oscillations decays more rapidly along the channel.

### 2.3 Quantitative Analysis of ESW Microscopy

**Figure 3:**
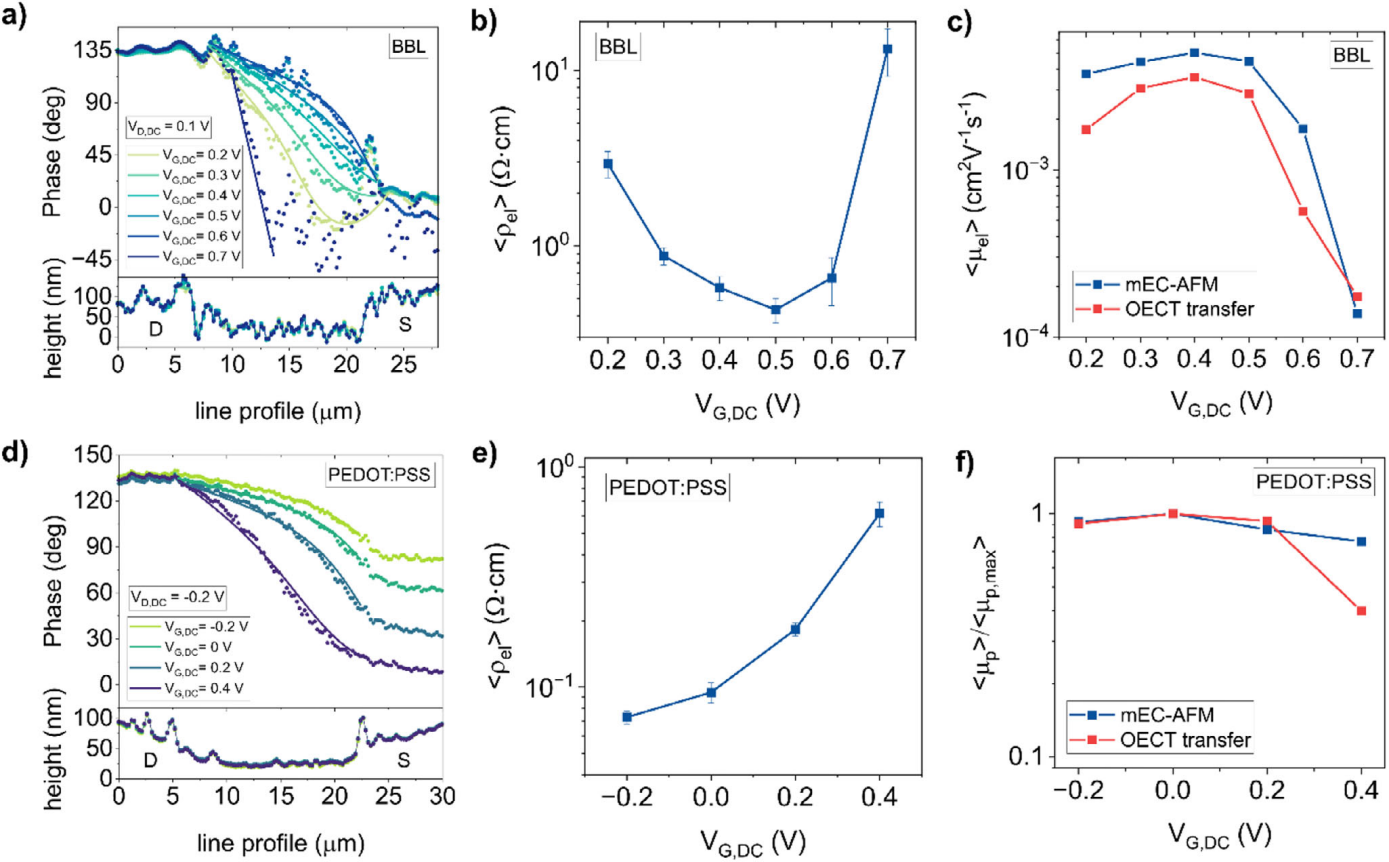
Quantitative analysis of electrochemical strain wave microscopy. mEC‐AFM phase profiles measured along BBL a) and PEDOT:PSS d) OECT channels with different DC gate potentials. The AC voltage modulation is added to the DC drain potential with frequency *f_BBL_* =1.7 kHz (a) and *f_PEDOT:PSS_* =3.1 kHz (d). Experimental values (indicated with dots) are fitted with Equation (3) (continuous line) to extract the propagation constant of the equivalent transmission line. Electronic resistivities of BBL b) and PEDOT:PSS e) channels as a function of the DC gate voltage. Max‐normalized electron c) and hole f) mobilities measured in BBL (c) and PEDOT: PSS (f) OECTs. Electrochemical strain wave microscopy data are compared with results calculated from OECT transfer characteristics.

Our findings in Figure 2 offer the first experimental evidence of ESW propagation in p‐type and n‐type OMIEC channels. Our next step is to establish how local strain amplitude and phase provide quantitative information on the charge transport properties of mixed‐conducting films. For this purpose, we developed a more systematic analysis measuring the profile of the ESW amplitude and phase along the OECT channel, while progressively changing the DC potentials applied to the gate and drain electrodes. The resulting traces are shown in **Figure** **3**a,d for BBL and PEDOT:PSS samples, respectively. (ESW amplitude profiles in Figure S1, Supporting Information). In addition to the phase signal, we show the channel morphology profile acquired in parallel. The local height spikes measured at the borders of the source and drain electrodes in Figure 3a,d are related to microfabrication defects, specifically residual metallic sidewalls underlying the OMIEC film remaining after the lift‐off process. Data acquired in correspondence of such defects are not considered in the quantitative analysis of ESW microscopy data. While the channel height profiles remain constant, the phase profiles show a particular dependence on the applied *V_G,DC_*. For BBL (Figure 3a) a constant phase is always obtained on the drain electrode. Upon entering the channel, the ESW phase drops. For small carrier density (*V_G,DC_* = 0.2 V), the decay is very rapid, indicating slow signal propagation. With increasing gate voltages, the phase drop is less pronounced. However, when large electronic carrier densities are accumulated in the channel, the phase profile experiences first a change in concavity (*V_G,DC_* = 0.6 V), and then again a rapid drop (*V_G,DC_* = 0.7 V). The results can be quantitatively analyzed by solving Equation (1) for each OECT operating condition. The boundary conditions were defined based on experimental observations (see Section T1, Supporting Information). At the drain electrode, the strain takes a value $\bar{s}$ *_0_*, thus we impose $\bar{s}\left(x=0\right)=\bar{s}$ *_0_*. At the same time, despite the source electrode acts as a ground for the applied AC modulation *V_D,AC_*, the strain phasor measured at *x* = *L* is not zero, but takes a finite value $\bar{s}$ *_L_* which is determined by the surface oscillations of the residual OMIEC layer overlapping the gold electrode. By imposing $\bar{s}\left(x=L\right)=\bar{s}$ *_L_*, Equation (1) is solved by

(5) $$\bar{s}\left(x\right)=\frac{\bar{s_{0}}\sinh\left[\gamma \left(L-x\right)\right]+\bar{s_{L}}\sinh\left(\gamma x\right)}{\sinh\left(\gamma L\right)}\;$$

where the propagation constant *γ* is defined by Equation (2). Despite measurements are affected by noise introduced by the rough morphology of the BBL layer, the acquired data are well‐reproduced by theoretical predictions (Figure 3a). The propagation constant resulting from the fit can thus be used to extract the electronic resistivity of the BBL channel. For this purpose, we used electrochemical impedance spectroscopy (see Figure S2, Supporting Information) to measure the volumetric capacitance of BBL and PEDOT:PSS, obtaining *c_v_* = 720 ± 40 Fcm^−3^ and *c_v_* = 40 ± 5 Fcm^−3,^ respectively, consistent with literature values.^[^ ^17^, ^57^ ^]^ Equation (2) was then used to calculate *ρ_el_* as a function of *V_G,DC_* as discussed in Section T4 (Supporting Information). Results reported in Figure 3b show a trend consistent with the OECT transfer curves presented in Figure 2c. The electronic resistivity initially decreases by increasing the doping level of the organic semiconductor through the applied gate voltage. However, when *V_G,DC_* > 0.6 V, *ρ_el_* sharply increases again, causing the rapid decay of the transistor channel current typical for the antiambipolar behavior of BBL OECTs.

Further analysis allows us to extract the average electronic mobility from our experimental data. In the linear regime, the carrier density $n=\frac{c_{v}}{e}\left(V_{G,DC}-V_{t}-\frac{V_{D,DC}}{2}\right)\;$ can be assumed as constant along the transistor channel, and the average electronic mobility is given by $\left\langle \mu_{n}\right\rangle =\frac{1}{ne\rho_{el}}$, where *e* is the elementary charge and *V_t_* is the threshold voltage. Average mobility values normalized by the peak mobility 〈μ_*n*,*max*_〉 obtained from microscopic mEC‐AFM measurements show an excellent agreement with macroscopic OECT transfer data (Figure 3c, Figure S3, Supporting Information). 〈μ_*n*_〉 exhibits a slight increase with gate voltage, reaching a maximum at 0.4 V, followed by a sharp decrease of over an order of magnitude as *V_G,DC_* approaches 0.7 V. Under this bias condition, the ESW microscopy data in Figure 3a show a low signal‐to‐noise ratio near the source region due to strong signal dissipation in the highly resistive channel. Nevertheless, the normalized mobility can be extracted reliably by fitting the first ten data points from the drain. These data points have sufficient signal quality, and fitting values remain consistent with both the ESW experimental trends and the macroscopic OECT results, confirming the reliability of the analysis.

The same study was repeated for the PEDOT: PSS‐based p‐type OECTs. Fitting experimental mEC‐AFM phase data acquired at different gate voltages with Equation (5) (Figure 3d) allows to calculate the electronic resistivity of the OMIEC thin film (Figure 3e). As PEDOT: PSS OECTs operate in depletion mode, *ρ_el_* increases with the applied gate voltage. However, the resistivity increase rate is smaller for high doping levels (*V_G,DC_* = ‐0.2 V), where the normalized hole mobility 〈μ_*p*_〉/〈μ_*p*,*max*_〉 resulting from both ESW microscopy and OECT transfer curves (Figure 3f) is smaller than the maximum value. Such observation is consistent with the transconductance drop measured in transfer characteristics at large carrier concentrations (*V_G,DC_* < 0 V in Figure 2b), which is typically observed in PEDOT: PSS OECTs.^[^ ^25^, ^27^ ^]^

### 2.4 Detection of Defects with ESW Microscopy

**Figure 4:**
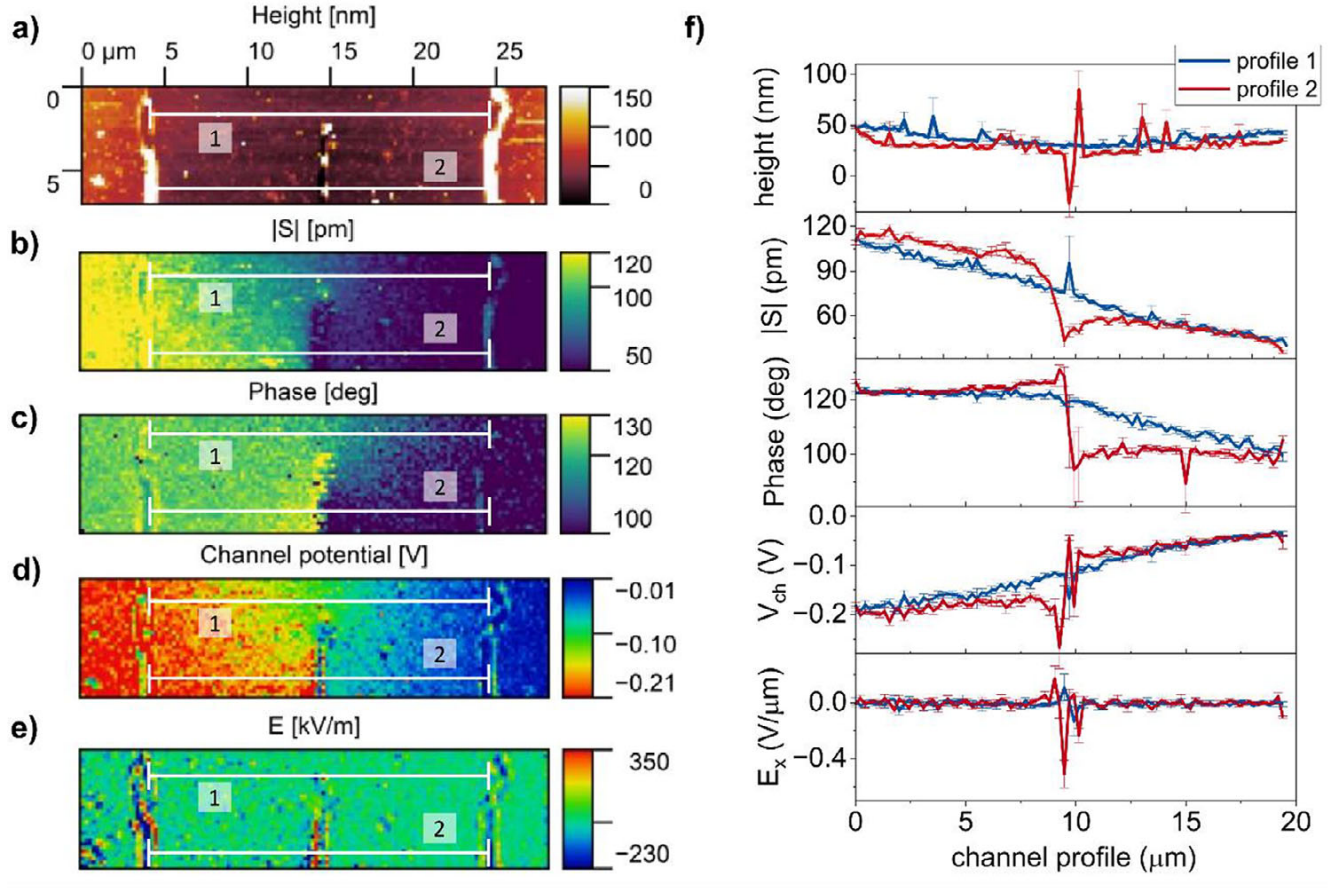
Local defects in the OECT channel alter the propagation of electrochemical strain waves. a) Morphological image of a PEDOT:PSS OECT channel where a vertical scratch was impressed using a wear‐resistant AFM probe. b) Resulting mEC‐AFM map of swelling amplitude and phase (c) acquired during device operation. d) Local channel potential and electric field e) calculated from ESW microscopy using the mathematical model in Section T5 (Supporting Information). f) Line profiles comparing mEC‐AFM data (blue line: unperturbed channel region, red line: channel region containing scratch defect).

mEC‐AFM maps acquired in Figures 2 and 3 are influenced by the morphology of the polymer layers. This effect is particularly evident for BBL OECTs (Figures 2d and 3a) due to the greater roughness of the OMIEC microstructure. Localized swelling clusters are observed in association with large film grains and are especially pronounced along the intersection between the OMIEC channel and the metallic electrodes, where the film morphology exhibits a sharp thickness variation (see Figure 3a). These observations suggest that ESW measurements are sensitive to inhomogeneities of the OMIEC layer and can be ultimately used to map local transport bottlenecks in the film. To verify such a hypothesis, we artificially introduced a microscopic defect in a PEDOT: PSS OECT channel (**Figure** **4**), and we studied its effect on ESWs propagation. DC potentials *V_D,DC_* = ‐0.2 V and *V_G,DC_* = 0 V were applied to the OECT to operate the transistor in the linear regime. A wear‐resistant AFM probe (NSC36‐Hard) was used to impress a vertical scratch (with width < 1 µm and length *l* = 5 µm) in the central region of the OMIEC thin film. The pattern is clearly visible from the sample morphology image in Figure 4a, also showing small PEDOT:PSS agglomerates composed by the material removed from the scratch line. mEC‐AFM images (Figure 4b,c) demonstrate that the vertical defect acts as a barrier for the strain wave propagation. The swelling distribution gradually decays along the channel in the upper portion of the film, which is morphologically uniform. However, a pronounced discontinuity appears in the region containing the scratch pattern. Line profiles extracted from both areas (Figure 4f) reveal that the spatial attenuation of the strain wave is predominantly concentrated around the scratch, where the swelling amplitude reaches a minimum and the phase exhibits a step‐like drop. These findings demonstrate how strain wave maps can pinpoint local defects that limit charge transport in operating OECTs.

At the same time, similarly to KPFM and scanning dielectric microscopy,^[^ ^58^, ^59^ ^]^ ESW microscopy can provide information on the OECT channel potential distribution *V_ch_(x)* (see Section T5 and Figure S4, Supporting Information for the full mathematical treatment). Results are reported in Figure 4d. In agreement with the DC bias applied to the transistor, *V_ch_(x)* assumes a constant voltage ≈−0.2 V on the drain electrode and reaches a value close to 0 V on the source contact. The channel potential linearly increases in the defect‐free region of the OECT channel, but shows a step‐like discontinuity (Figure 4f) in the presence of the vertical scratch. Consistently, the horizontal component of the electric field $E_{x}=-\frac{dV_{ch}\left(x\right)}{dx}$ along the channel (Figure 4e) assumes a constant value of 9 ±2 mV µm^−1^ in the linear operating region of the device, but locally spikes on the introduced defect.

## 3 Discussion

In this work, we introduce electrochemical strain wave microscopy as a novel technique to investigate local transport processes in operating OECTs. The technique is based on modulated atomic force microscopy experiments under electrochemical control and maps the amplitude and the phase of electrochemical strain waves injected at the drain electrode into OECT channels. By performing in‐operando experiments on different n‐type and p‐type OECT devices, we probe how strain wave propagation is influenced by the doping level as controlled by the gate bias. Systematic measurements are interpreted through a quantitative theory based on a transmission line model and are consistent with macroscopic OECT transport measurements. The findings allow to extract the local electronic carrier mobility of the OMIEC materials independent of contact effects. Experimental results also demonstrate how ESW microscopy can determine local defects causing charge transport bottlenecks, or map the local channel potential, obtaining results analogous to KPFM experiments in organic field effect transistors.^[^ ^58^ ^]^

Our technique is applicable to studying charge transport in OECTs based on different classes of mixed ionic–electronic conductors. Key experimental considerations include the measurement timescale and the amplitude dissipation of the ESWs. In materials with low electronic conductivity, only low‐frequency ESWs propagate sufficiently to be detected, as quantified by the propagation constant in Table T4 (Supporting Information). Furthermore, the measurement timescale can be constrained by ion transport through the electrolyte as expressed by the RC time constant in devices with thick or large‐area films (large C) and low conductive electrolyte (large R). For example, to obtain an image with 128x128 pixels in 30 min, a time of less than 100 ms is available per image pixel. To record the amplitude and phase in such a time interval, the ESW should have a frequency higher than 500 Hz. If this frequency range exceeds significantly the RC time constant of the electrochemical device, no significant ion accumulation in the OMIEC would occur, and hence the signal amplitude would be too small to measure. To further place our results in the context of existing characterization methods, Section S7 (Supporting Information) provides a comparison table summarizing the main performance metrics of ESW microscopy relative to other scanning probe techniques applied to OMIEC characterization.

The findings obtained in this work hold two major advancements for OECT research and OMIEC material science. First, this work offers a nanoscale insight into dissipative charge transport processes governing the OECT operation. The spatial attenuation and dispersion of the ESWs propagating in the channel are caused by intrinsic mixed ionic and electronic conduction processes in the OMIEC channel. Direct measurements of the propagation constant at different doping levels provide a quantitative metric to benchmark both the signal propagation speed and the energetic efficiency of OECT materials and can guide the development of novel OMIEC formulations to enhance the performance of bioelectronic sensors.^[^ ^49^ ^]^ In parallel, ESW microscopy enables the identification of extrinsic factors such as local inhomogeneities, defects, and fabrication issues that further limit device performance and stability. Second, from this study we highlight significative differences between BBL‐ and PEDOT: PSS‐based OECTs in terms of device operation. N‐type BBL‐based OECTs exhibit an antiambipolar behavior, which is currently being investigated to develop organic electrochemical neurons for neuromorphic computing.^[^ ^60^ ^]^ Our analysis provides a quantitative explanation of such an effect in terms of a carrier density dependent mobility causing the negative device transconductance at high density levels. In contrast, PEDOT: PSS OECTs do not display this behavior; instead, they demonstrate faster signal propagation and lower energy dissipation as indicated by the smaller propagation constant measured in our experiments, making them more suitable for bioelectronic recording applications.^[^ ^61^ ^]^ These findings underscore that, while the term “OECT” is generally used to refer to devices based on both materials, it may be ambiguous regarding specific differences in device physics. Different electronic and ionic transport properties arising from different active materials necessitate more material‐specific analysis to accurately test device functionality and performance. Nanoscale measurements of OECT transport with electrochemical strain wave microscopy are expected to provide further contributions in this direction.

## 4 Experimental Section

### PEDOT: PSS OECTs Fabrication

Glass substrates (50×25 mm^2^) were cleaned by sonication in acetone/isopropanol/distilled water baths. After a dehydration step (10 min at 110° C), the Microposit S1818 positive photoresist was spin coated (4000 rpm for 60 s) and annealed at 110 °C for 1 min. Metallic contacts were patterned through direct writing lithography by using the ML3 Microwriter (from Durham Magneto Optics). The photoresist was developed with Microposit MF‐319 developer. Then, 7 nm of chromium and 30 nm of gold were deposited by thermal evaporation. Samples were immersed in acetone for 4 h for photoresist lift‐off. Metallic contacts were encapsulated with the mr‐DWL 5 negative photoresist (from Micro Resist Technology). The resin was spin coated at 3000 rpm for 30 s and annealed at 100 °C for 2 min. After laser exposure, samples were baked at 100 °C for 2 min and relaxed for 1 h at room temperature. Development was performed with the mr‐Dev 600 developer (Micro Resist Technology), and the resist was finally hard‐baked at 120 °C for 30 min. A layer of S1818 was spin‐coated (2000 rpm for 60 s) for the photolithography of the PEDOT: PSS channel. After development, substrates were treated with air plasma (15 W for 4 min) and the PEDOT: PSS solution (94% PEDOT: PSS (Heraeus, Clevios PH1000), 5% of ethylene glycol (EG) (Sigma–Aldrich), 1% of 3‐glycidoxypropyltrimethoxysilane (GOPS), and 0.25% of 4‐dodecylbenzenesulfonicacid (DBSA)) was spin coated at 3000 rpm for 9 s. The resulting film thickness was (85 ±10) nm. The samples were subsequently annealed at 120 °C for 1 h, and S1818 was finally lifted‐off after 4 h in isopropanol.

### BBL OECTs Fabrication

BBL was synthesized following previous reports.^[^ ^62^ ^]^ Four‐inch glass wafers were initially cleaned by sequential sonication in a 2% (v/v) industrial surfactant solution (Micro‐90), acetone, and isopropyl alcohol, followed by drying under a nitrogen stream. Subsequently, electrodes comprising 5 nm of chromium and 50 nm of gold were thermally evaporated and patterned via standard photolithography and wet etching procedures. A 1‐µm‐thick layer of PaC was then deposited as an insulating layer to separate the metal electrodes from the electrolyte. To improve adhesion, silane A‐174 was applied prior to PaC deposition. An anti‐adhesive layer consisting of 2% Micro‐90 surfactant was spin‐coated onto the PaC surface, followed by the deposition of a 2‐µm‐thick sacrificial PaC layer. A 5‐µm‐thick positive photoresist was then spin‐coated, exposed, and developed to define the channel and contact pad regions. This patterned photoresist served as a protective mask during plasma reactive ion etching (RIE), which was conducted under the following conditions: 150 W RF power, 500 sccm O_2_, 100 sccm CF_4_, for 380 s. The etching step selectively removed the exposed organic materials, including the photoresist and PaC, to reveal the OECT channel and contact pads, while preserving PaC coverage elsewhere. After cleaning the wafer with acetone, a 20‐nm‐thick BBL layer was deposited by spin‐coating a BBL solution (2.5 mg mL^−1^ in MSA) at 1000 rpm for 60 s, followed by immersion in water and drying with nitrogen. Finally, the BBL layer was patterned using a sacrificial PaC lift‐off process.

### Electrical Measurements

DC characteristics of the OECTs were carried out with the Keysight 2912A source‐measure unit (SMU), using a Ag/AgCl wire as the gate electrode. The acquired data were analyzed to set the working point of the transistor during mEC‐AFM imaging experiments. AC measurements, including mEC‐AFM spectroscopies (Figures S5 and S6 and Section T2, Supporting Information) and electrochemical impedance spectroscopy (Section T6, Supporting Information), were performed with the MFLI lock‐in amplifier (from Zurich Instruments).

### mEC‐AFM Imaging Experiments for ESW Microscopy

Atomic force microscopy (Park System's NX10 AFM) was performed in liquid, using 0.1 m PBS as electrolyte and an Ag/AgCl wire as reference electrode. A sinusoidal oscillation *V_D,AC_* (amplitude of 100 mV) with desired frequency *f* was applied to the drain electrode to generate electrochemical strain waves. DC potentials *V*_D,DC_ and *V*_G,DC_ were applied to the drain and gate electrodes, respectively, to enable the OECT operation. The cantilever sensitivity *s_c_* = 129±5 V µm^−1^ of NSC36 probes was measured through repeated force‐distance spectroscopies on the glass substrate of the samples (see ref.[45] for full experimental details). Multichannel images were acquired on the OECT channels by operating the Park NX10 AFM in PinPoint mode. The operating frequencies *f_im_* for ESW microscopy experiments were chosen to optimize signal to noise ratio while maintaining imaging times sufficiently small. Despite higher frequencies were advantageous as they reduce the acquisition time and shift the signal from low frequency noise, these should not exceed the RC time constant of the OECT, as this causes a strong reduction in signal amplitude (see ref. [44] for full details). The basic PinPoint parameters were set as follows. For PEDOT: PSS OECTs: *f_im_* = 3.1 kHz, cantilever approach/retract speed = 20 µm s^−1^, force set point = 12 nN, z‐servo duration = 4 ms, average time = 1 ms, pixel‐to‐pixel move time = 5 ms. For BBL OECTs: *f_im_* = 1.7 kHz, cantilever approach/retract speed = 20 µm s^−1^, force set point = 15.5 nN, z‐servo duration = 7 ms, average time = 1.75 ms, pixel‐to‐pixel move time = 5 ms. In both cases, the z‐scanner motion range was restricted to 3 µm. During PinPoint operation, the amplitude and phase of the local OMIEC swelling were determined from the PSPD‐signal with an external lock‐in amplifier (MFLI Zurich Instruments) when the tip was held in contact. Both values were saved as additional image channels for each PinPoint pixel using the auxiliary input channels of the NX10 system.

## Conflict of Interest

The authors declare no conflict of interest.

## Supporting information



Supporting Information

## Data Availability

The data that support the findings of this study are available from the corresponding author upon reasonable request.
