# Supplementary material for: Investigation of Transport in OECTs with Electrochemical Strain Wave Microscopy
Source: Small. 2025 Nov 14;21(52):e10345. doi: 10.1002/smll.202510345 (PMC12747622; doi:10.1002/smll.202510345)
Supplement: Supplementary file 1 — Supporting Information [file SMLL-21-e10345-s001.docx]

**Supporting Information**

**Figures**


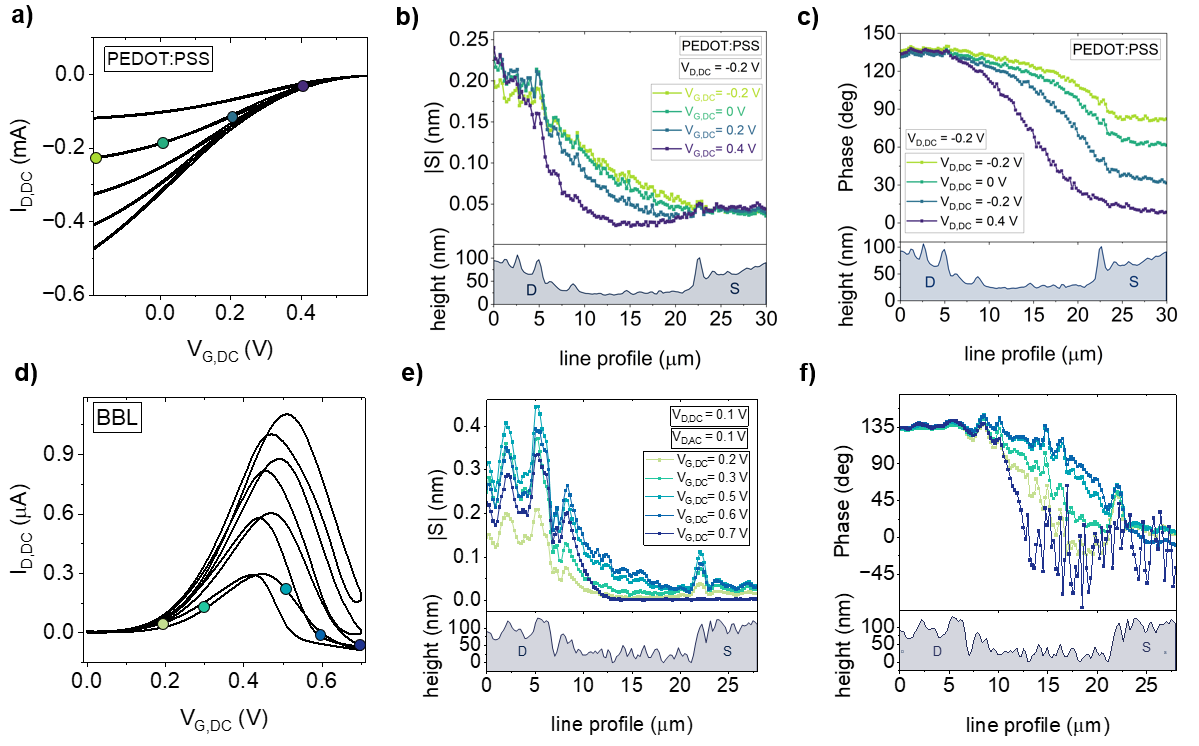


**S1:** Operando electrochemical strain wave microscopy linescans in OECTs during operation. a) Transfer characteristics of a PEDOT:PSS OECT, indicating the transistor working point investigated with mEC-AFM line profiles. Local swelling amplitude (b) and phase (c) measured by varying the applied DC gate potential. The AC voltage modulation is superimposed to the DC drain potential with frequency f=3.1 kHz. d) Transfer characteristics of a BBL OECT, indicating the transistor working point investigated with mEC-AFM line profiles. Local swelling amplitude (e) and phase (f) measured by varying the applied DC gate potential. The AC voltage modulation is superimposed to the DC drain potential with frequency f=1.7 kHz. Additional information in Supp. Inf. T3.


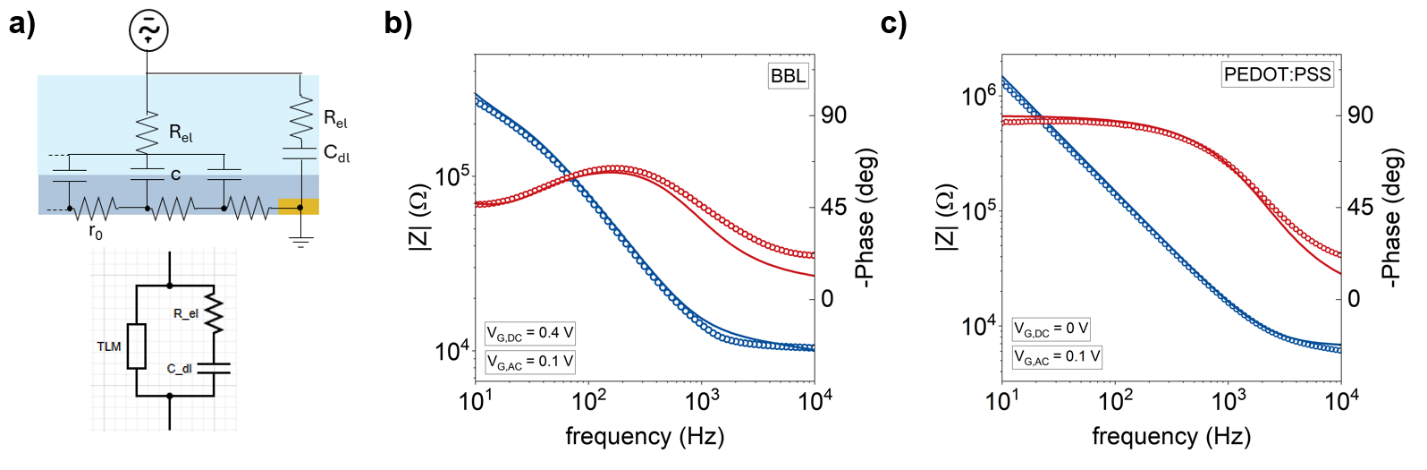


**Figure S2:** Electrochemical impedance spectroscopy (EIS) of OMIEC channels. a) Equivalent circuit model and Bode plot for BBL (b) and PEDOT:PSS (c) layers. Experimental data (indicated with circles) show a good agreement with fitting results (continuous line). Additional information in Supp. Inf. T6)


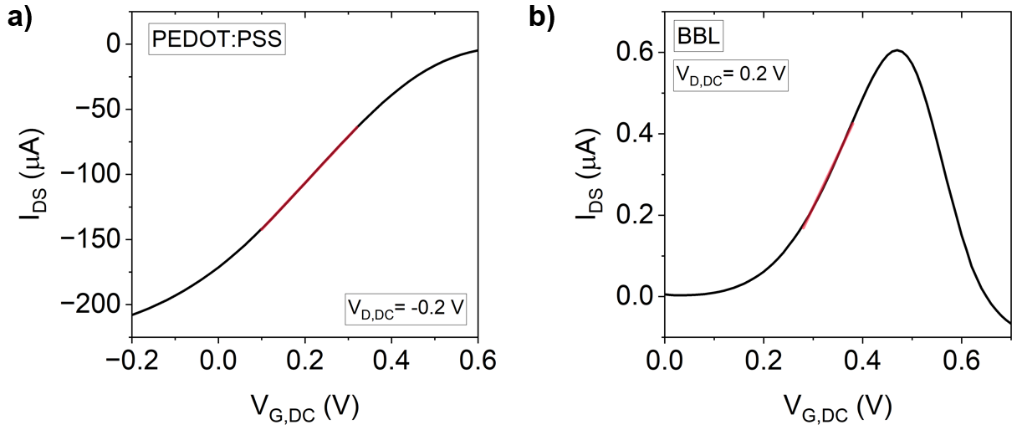


**Figure S3:** DC transfer characteristics of PEDOT:PSS (a) and BBL (b) OECTs. The linear operation regime of the transistors was fitted with the Bernards-Malliaras model to extract the threshold voltage. Additional information in Supp. Inf. T7.


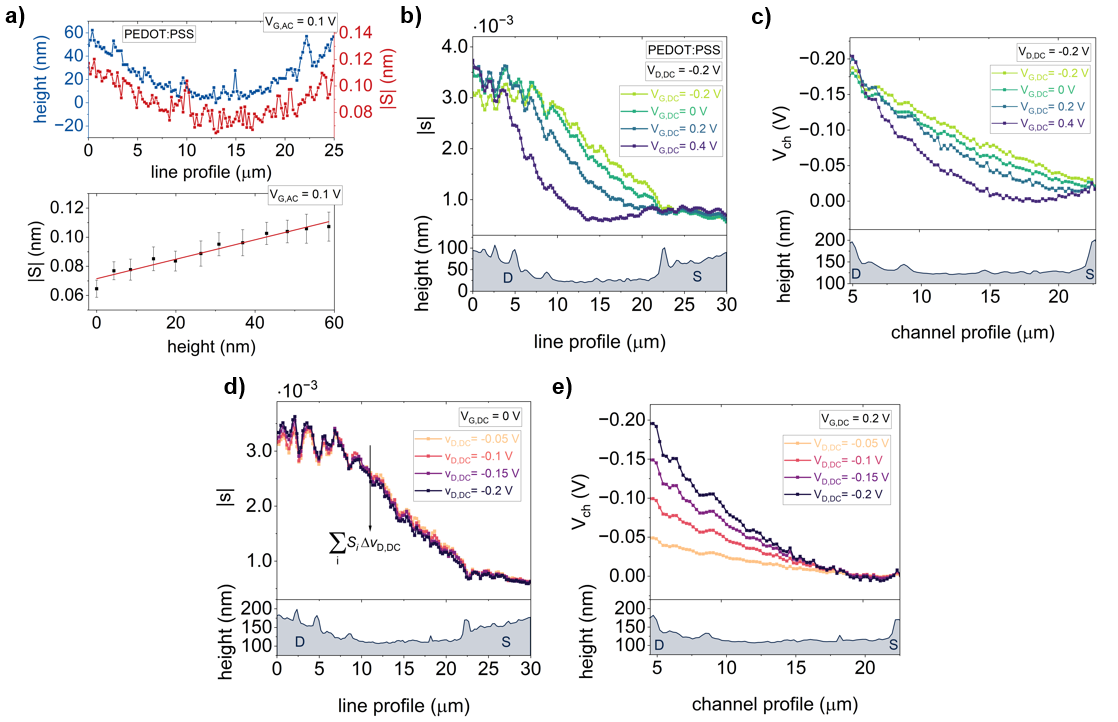


**Figure S4:** In-operando nanoscale determination of the local channel potential in OECTs with electrochemical strain wave microscopy in PEDOT:PSS OECTs. a) Local correspondence and statistical correlation between local thickness and swelling of the PEDOT:PSS layer measured by applying and AC modulation to the gate electrode. From the correlation plot, it is possible to evaluate the effective thickness of the OECT channel contributing to swelling. b) Local strain amplitude measured by varying the applied DC gate potential. The AC voltage modulation is superimposed to the DC drain potential with frequency f=3.1 kHz. c) Local OECT channel potential calculated at different OECT working points by varying the DC gate voltage. d) Swelling curves measured at different DC drain potential. From their discrete integration, it is possible to approximate the integral in Equation S19. e) Local OECT channel potential calculated at different OECT working points by varying the DC drain voltage. Additional information in Supp. Inf. T5


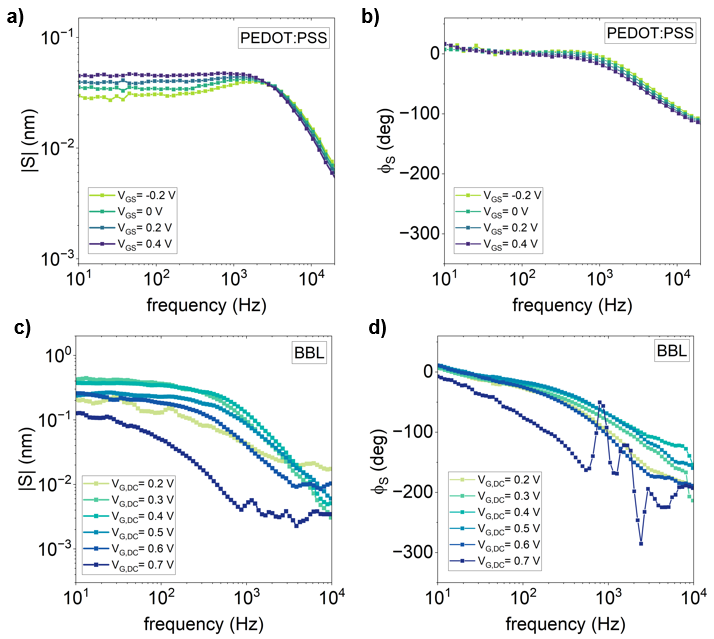


**Figure S5:** mEC-AFM spectroscopies of PEDOT:PSS and BBL layers measuring the amplitude (a, c) and the phase (b, d) of the OMIEC swelling (acquired at the center of the channels) at different DC gate voltages. Additional information in Supp. Inf. T2.


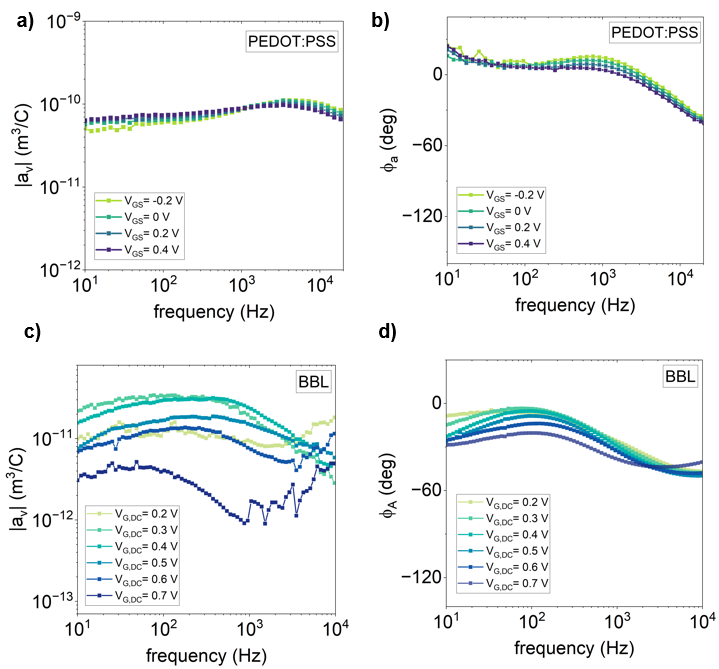


**Figure S6:** Volumetric actuation function amplitude (a, c) and phase (b, d) for PEDOT:PSS and BBL samples. Additional information in Supp. Inf. T2.


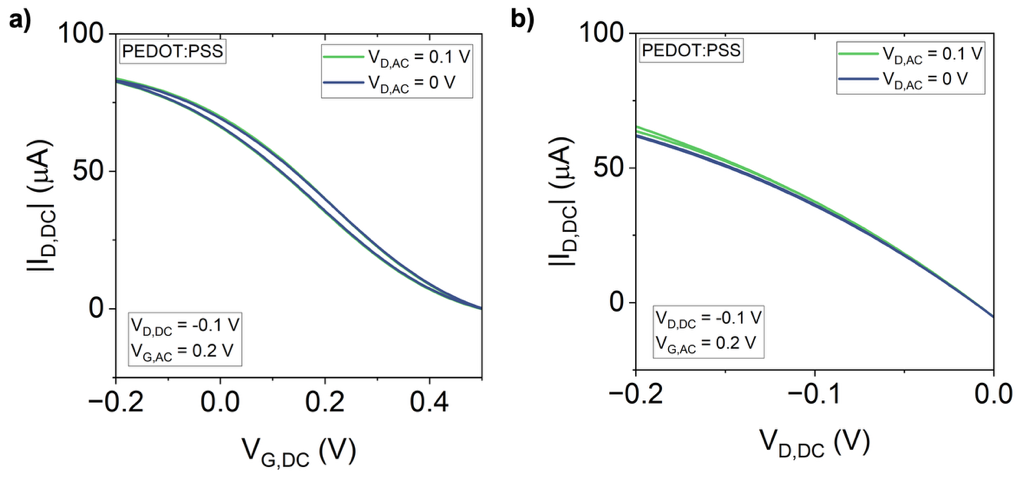


**Figure S7:** OECT transfer (a) and output (b) characteristics acquired in DC conditions by applying and not applying the AC modulation to the drain electrode.

**Tables:**

| **Technique** | **Operational conditions** | **Spatial resolution** | **Operational frequency** | **Quantitative capability** |
| --- | --- | --- | --- | --- |
| This work (mEC-AFM^[1]^) | In liquid, *operando* | 20-30 nm | 500 Hz – 10 kHz | Yes: *n, V_ch_* |
| ESM (electrochemical strain microscopy)^[2]^ | In air/liquid, *operando* | 20-30 nm | 1 kHz – 10 kHz | Yes:* $\mu_{ion}$ |
| SECM (scanning electrochemical microscopy)^[3]^ | In liquid, *operando* | 1-10 µm | 0.1 – 10 Hz | Yes:  *V_ch_* |
| SDM (scanning dielectric microscopy)^[4,5]^ | In air/liquid, *operando* | 20-30 nm | up to MHz | Yes:* $\sigma$*, V_ch_* |

*measurements require interpretation with complex models

**Table S7:** Comparison between established scanning probe techniques used to characterize OECTs during operation. In the last column, n indicates the carrier density, V_ch_ the channel potential, $\mu_{ion}$ the ionic mobility, $\sigma$ the electronic conductivity of the OMIEC channel.

**T1. Transmission line theory for electrochemical strain waves**

Recent works studying signal propagation in electrolyte-gated OMIEC channels demonstrate that a material consisting of mixed conductor volume elements can be accurately represented for small-signal analysis by an equivalent circuit model corresponding to an electrical transmission line.^[6]^ In the frequency domain, the propagation of an electrical potential *V(x,t)* = Re{ $\bar{V}$*(x)*$e^{j\omega t}$} along the line can be turned into a single variable differential equation for the voltage phasor $\bar{V}$*(x)*:^[7]^

$$\frac{d^{2}\bar{V}\left( x \right)}{dx^{2}}-\gamma^{2}\bar{V}\left( x \right)= 0 (S1)$$

where the propagation constant *γ* is defined in Eq. 2 as the ratio between the horizontal impedance and the vertical (or characteristic) impedance of the line (see ref. ^[6]^ for the full mathematical treatment). The propagating AC potential wave alters the local charge distribution through the injection/ejection of mobile cations, causing dynamic volume changes in the channel that propagate as an electrochemical strain wave (ESW) along with the electrical signal. Given an OMIEC channel with length *L*, width *W*, thickness *t_h_,* and volumetric capacitance *c_v_,* we define the local electrochemical strain as

$$\bar{s}\left( x \right)=\frac{\bar{S}\left( x \right)}{t} (S2)$$

where $\bar{S}\left( x \right)$ is the swelling of the OMIEC film measured by mEC-AFM experiments. $\bar{s}\left( x \right)$depends on the amount of injected ionic charge density by ^[1,8]^

$$\bar{s}\left( x \right)=a_{v}\cdot\bar{\rho}\left( x \right) (S3)$$

where $\bar{\rho}\left( x \right)=\bar{Q}(x)/(WLt_{h})$ is the charge density phasor (expressed in C/m^3^) and *a_v_* indicates the volumetric actuation transfer function (see Supp. Inf. 3), which can be determined experimentally from spectroscopies in Figure S5 and S6. The charge density phasor can be expressed as a function of the potential along the line (channel potential) as:^[8]^

$$\bar{\rho}\left( x \right)=c_{V}\bar{V}\left( x \right) (S4)$$

giving

$$\bar{s}\left( x \right)=a_{v}c_{V}\bar{V}\left( x \right) (S5)$$

The volumetric actuation function *a_v_* is determined by the ionic hydration radius of the injected ions, water uptake and morphological changes in the polymer. *a_v_* can be assumed to be independent from the position along the line. Thereby, for small signals, the swelling phasor is directly proportional to the voltage phasor, and Eq. S1 can be written in terms of the OMIEC strain as

$$\frac{d^{2}\bar{s}\left( x \right)}{dx^{2}}-\gamma^{2}\bar{s}\left( x \right)= 0 (S6)$$

which is solved by

$$\bar{s}\left( x \right)= \bar{s_{0}^{+}}e^{-\gamma x}+\bar{s_{0}^{-}}e^{\gamma x} (S7)$$

where the "+" and "-" superscripts label respectively forward and backward travelling strain waves.

Two different sets of boundary conditions are generally imposed for Eq. S6 to determine the unknown coefficients $\bar{s_{0}^{+}}$ and $\bar{s_{0}^{-}}$ in Eq. S7. For floating channels

$$\left\{ \begin{aligned} \bar{s}\left( x=0 \right)=\bar{s_{0}} \\ \frac{d\bar{s}}{dx}\left( x=L \right)=0 \end{aligned} (S8) \right.$$

we obtain

$$\bar{s}\left( x \right)=\frac{\bar{s_{0}}}{\cosh(\gamma L)}\cosh\left[ \gamma(L-x) \right] (S9)$$

For grounded channels

$$\left\{ \begin{aligned} \bar{s}\left( x=0 \right)=\bar{s_{0}} \\ \bar{s}\left( x=L \right)=0 \end{aligned} (S10) \right.$$

we obtain

$$\bar{s}\left( x \right)=\frac{\bar{s_{0}}}{\sinh(\gamma L)}\sinh\left[ \gamma(L-x) \right] (S11)$$

As the source electrode acts as a ground for the applied AC modulation *V_D,AC_*, we used Eq. S11 to model electrochemical strain wave propagation in the conceptual scheme in Figure 1. However, in experimental measurements the swelling phasor measured at *x*=*L* is not zero, but assumes a finite value $\bar{S_{L}}$ determined by the surface oscillations of the OMIEC layer overlapping the gold electrode. For this reason, experimental measurements in Figure 5 were fitted imposing experimental boundary conditions

$$\left\{ \begin{aligned} \bar{s}\left( x=0 \right)=\bar{s_{0}} \\ \bar{s}\left( x=L \right)=\bar{s_{L}} \end{aligned} (S12) \right.$$

giving

$$\bar{s}\left( x \right)=\frac{\bar{s_{0}}\sinh\left[ \gamma(L-x) \right]+\bar{s_{L}}\sinh\left( \gamma x \right)}{sinh(\gamma L)} (S13)$$

which is formally analogous to a grounded channel, but contains an extra term corresponding to a (small amplitude) back-propagating strain wave from the source channel.

**T2. mEC-AFM spectroscopies for mixed conductors**

Multidimensional mEC-AFM spectroscopies simultaneously acquire the AC voltage, current and swelling phasors to study the electroswelling properties of OMIEC materials at different frequencies. mEC-AFM spectroscopies are performed by shorting the source and the drain electrodes in Figure 2a and applying an AC modulation the Ag/AgCl gate with respect to the OMIEC layer. In this configuration, injection/extraction of ionic charge carriers occurs simultaneously in all regions of the OMIEC thin film causing the swelling of the polymer $\bar{S}(\omega)$, which is measured at the center of the OECT channel. Spectroscopic measurements are acquired by sweeping the frequency of the AC signal from 10 Hz to 10 kHz at different DC gate bias. Experimental data show how for both mixed conductors the swelling amplitude (Figure S5a and S5c) has a plateau in the low frequency regime with a stable phase of 0° (Figure S5b and S5d). Then, after a critical frequency, the phase shift reduces, and the swelling amplitude decreases with increasing frequency. These observations indicate that the local height of the film increases (by a value of |*S*|) when the OMIEC layers are negatively charged and cations are injected into the film bulk to keep electroneutrality (see also Figure S6).^[1]^ While mEC-AFM spectroscopies acquired with PEDOT:PSS samples show a slight dependence on the applied gate voltage, BBL data indicate a larger impact of the material doping level. In particular, at high carrier densities (*V_G,DC_* =0.7 V) the swelling process is significantly slower and has smaller extent.

Phasors measured in mEC-AFM spectroscopies can be related using the electrochemical impedance $Z=\bar{V}/\bar{I}$ and volumetric actuation transfer function $a_{v}=\bar{s}\left( x \right)/\bar{\rho}\left( x \right)$ defined according to Eq. S3. $a_{v}$ reveals how the morphology of the soft polymer matrix changes through the injection of charge and is determined by the size (or size distribution) of the hydrated ions injected in the film (see ref ^[1,8]^ for a detailed description). Spectra of $a_{v}$ for PEDOT:PSS and BBL channels acquired using the setup in Figure 2a are reported in Figure S2.2. The actuation function amplitude for PEDOT:PSS samples is almost independent from the DC gate voltage (Figure S2.2a), while the electroactuation properties of BBL are affected by the doping level of the organic semiconductor (Figure S6c). At the same time, the actuation function phase has a similar behavior for both materials (Fig. S6b and S6d). *ϕ_A_* slightly drops at high frequencies but plateaus at low frequency reaching values of about 0°. Such evidence indicates how the volume of both OMIEC films increases when negative electronic charges/positive cations are injected in the film. The measurements therefore confirm the proportionality between $\bar{s}$ and $\bar{V}$ for small signals (< 100 mV) for BBL and for PEDOT:PSS even for larger signals.

**T3. Operando electrochemical strain wave microscopy linescans in OECTs**

Findings in Figure 2 offer the first experimental evidence of ESW propagation p-type and n-type OMIEC channels. To establish how local strain amplitude and phase provide quantitative information on the charge transport properties of mixed-conducting films, we systematically measured the profile of the ESW amplitude and phase along the OECT channel, while progressively changing the DC potentials applied to gate and drain electrode. The resulting traces in Figure S1 map the evolution of the swelling amplitude distribution in the channel when the electronic conductivity of the OECT channel is gradually changed. The AC modulation was applied to the drain electrode (on the left) with a frequency *f* = 3.1 kHz in PEDOT:PSS (Figure S1 b-c) and *f* = 1.7 kHz in BBL samples (Figure S1 e-f). Traces measured in PEDOT:PSS OECTs (figure S4b and S4c) map the evolution of the swelling distribution in the channel when the electronic conductivity of the OECT channel gradually decreases. The corresponding transfer characteristics of the transistor are reported in Figure S1a. Data are compared with the local morphology of the PEDOT:PSS layer. The acquired swelling amplitude profiles (Figure S1b) assume a different value on the OECT drain electrode depending on the applied gate potential. Curves then intersect at the initial point of the OECT channel (position x ≈ 7 µm) and drop along the horizontal direction until the source electrode, where a constant swelling is measured for every trace. The rate of decay of the swelling amplitude |S| in the channel depends on the OECT working point. When the transistor channel is highly doped (*V_G,DC_* = -0.2 and 0 V), |*S*| decreases from drain to source with an (approximatively) constant slope. On the other hand, for low conductive channels (*V_G,DC_* = 0.4 V) the swelling signal drops rapidly close to the drain. The corresponding phase of the swelling oscillations (Figure S1c) assumes a constant value on the drain electrode, but then decreases more rapidly when the PEDOT:PSS layer is gradually de-doped.

mEC-AFM traces acquired in BBL OECTs (figure S1e and S1f) map the evolution of the swelling distribution in the channel when the electronic conductivity of the OECT channel gradually increases. Swelling amplitude curves (Fig. S1e) assume a maximum value on the drain and electrode and progressively drop along the channel. The strain extent is initially small for low carrier density (*V_G,DC_* = 0.2 V), progressively increases as the gate voltage increases, and finally drops at high doping level (*V_G,DC_* = 0.7 V) consistently with phase data (Figure S1f) extensively discussed in the main text of the manuscript.

**T4. Propagation constant for OECT channels**

The propagation constant of the OECT channel can be calculated considering the equivalent circuit model in Fig. 1c. In the horizontal direction, the transmission line considers only the electronic carrier path described by the electronic resistance per unit length *r_0_* (measured in Ω/m). This is justified by the electronic conductivity of the OMIEC channel exceeding by several orders of magnitude its ionic counterpart^[9]^. In the vertical direction, boundary conditions are determined by the inert substrate and by the electrolyte bath acting as a selective contact for ionic carriers. For thin films we assume that the electronic path in the *y*-direction is at equipotential, and we neglect its electronic resistances. The electronic path is coupled through the volumetric capacitance to the ionic transport path in the y-direction characterized by the ionic resistance $r_{ion}$ of the volume element. Thereby, the characteristic impedance *z_0_* of the vertical line (measured in Ω*m) can be modeled with an RC series circuit as shown in Fig. 1c leading to the transmission line constants^[6]^

$$r_{0}=\frac{\rho_{el}}{Wt_{h}}; z_{0}=\frac{1}{j\omega c_{v}Wt_{h}}+\frac{\rho_{ion}t_{h}}{W} (S14)$$

giving

$$\gamma=\sqrt{\frac{r_{0}}{z_{0}}}=\sqrt{\frac{j\omega c_{v}\rho_{el}}{1+j\omega c_{v}\rho_{ion}{t_{h}}^{2}}} \left( S15 \right)$$

Recent studies on organic mixed conductors demonstrate that the ionic conductivity of OMIEC channels is only slightly modulated by the electrochemical processes driving the OECT operation.^[6,10]^ Thereby, for the purpose of this work we can neglect the resistive effects related to ionic transport within the film and consider only ionic conduction in the electrolyte (modeled with the resistance *R_S_* in Figure 1). The propagation constant becomes

$$\gamma=\sqrt{\frac{r_{0}}{z_{0}}}=\sqrt{j\omega c_{v}\rho_{el}} \left( S16 \right)$$

which corresponds to the low-frequency limit of Eq S15.^[6]^

**T5. Operando determination of the OECT channel potential with electrochemical strain wave microscopy**

The objective of this section is to determine the channel potential *V(x)* from the phasor of the surface strain oscillation $\bar{s}$ measured at different positions *x* in the channel. The phasor depends also on the frequency *ω* and will be determined by the DC voltages applied to gate and drain electrode (*V_D,DC_* and *V_G,DC_*). In addition, we have the phasor describing the AC voltage applied to the drain electrode $\bar{V_{D,AC}}$ to generate the strain wave. For linear response, we can state that the ratio of the two phasors corresponds to the derivative of the local strain $\bar{s}$(*x*) with respect to drain voltage *V_D_*:

$$\frac{\bar{s}(x,V_{D,DC},\omega)}{\bar{V_{D,AC}}}=\frac{ds\left( x \right)}{dV_{D}} \left( S17 \right)$$

As discussed in Supp. Inf. S1, the local electrochemical strain is directly proportional to the charge density $d\bar{s}\left( x \right)= a_{v} d\bar{\rho}\left( x \right)$, where *a_v_* denotes the volumetric actuation function. Similar, the charge density depends linearly on the local channel potential: $d\rho\left( x \right)=c_{v}dV\left( x \right)$. Therefore, we obtain

$$\frac{\bar{s}(x,V_{D,DC},\omega)}{\bar{V_{D,AC}}}=a_{v}(\omega) c_{v} \frac{dV\left( x \right)}{dV_{D}} \left( S18 \right)$$

By integration we obtain:

$$V\left( x \right)=\int_{V\left( x \right)=0}^{V(x)} dV\left( x \right)=\frac{1}{a_{v}(\omega) c_{v}}\int_{V_{D,DC=0}}^{V_{D,DC}} \frac{\bar{s}(x,V_{D,DC},\omega)}{\bar{V_{D,AC}}(\omega)}dV_{D} \left( S19 \right)$$

In the linear regime, *s(x)* does not depend on *V_D,DC_* and we can simplify:

$$V\left( x \right)=\frac{1}{a_{v}(\omega) c_{v}}\frac{\bar{s}(x,V_{D,DC},\omega)}{\bar{V_{D,AC}}}V_{D,DC} \left( S20 \right)$$

In the saturation regime, we can solve the integral numerically:

$$V\left( x \right)\approx\frac{1}{{a_{v}(\omega)c}_{v}\bar{V_{D,AC}}\left( \omega\right)} \sum_{i} \bar{s}_{i}\left( x,V_{D,DC},\omega\right)\cdot\Delta V_{D,DC} \left( S21 \right)$$

where $\bar{s}_{i}(x,\omega)$ are swelling phasors measured in the range [0, *V_D,DC_*] with step $\Delta V_{D,DC}.$

Finally, the electric field along the channel *E_x_* is given by

$$E_{x}\left( x \right)=-\frac{dV}{dx}\left( x \right)=-\frac{1}{a_{v}(\omega)c_{v}\bar{V_{D,AC}}(\omega)}\int_{V_{D,DC=0}}^{V_{D,DC}} \frac{d\bar{s}}{dx}(x,V_{D,DC},\omega)dV_{D} \left( S22 \right)$$

which in linear regime becomes

$$E_{x}\left( x \right)=-\frac{V_{D,DC}}{a_{v}(\omega)c_{v}\bar{V_{D,AC}}(\omega)}\frac{d\bar{s}}{dx}(x,V_{D,DC},\omega) \left( S23 \right)$$

We looked for the experimental verification of our model considering PEDOT:PSS data due to their high signal-to-noise ratio, but the underlying arguments apply to any mixed conducting channel. mEC-AFM measurements provide information on both the local swelling *S(x)* and height *h(x)* of the OECT channel. Determining the electrochemical strain $\bar{s}\left( x \right)=\bar{S}(x)/t_{h}(x)$ requires knowledge on the effective thickness *t_h_(x)* contributing to the polymer swelling*.* This can be determined from mEC-AFM line-scans by applying an AC modulation to the gate electrode.^[1]^ Data traces measured at different OECT working points show a strong correspondence between local swelling and PEDOT:PSS channel topography as reported in Figure S4a, presenting results obtained at *f = 3.1* kHz, *V_D,DC_ =* -0.2 V and *V_G,DC_* = -0.2 V. Such evidence is rationalized studying the statistical correlation between the two quantities, which can be interpreted with a linear model in the form *S(x) = α*h(x)+β*. By considering the intercept *β* = (0.071±0.008) nm as the contribution on the local swelling provided by the underlying PEDOT:PSS layer, the effective thickness of the channel for every OECT working point can be calculated as *t_h_(x) = h(x) + β/α.* The resulting electrochemical strain on the OMIEC channel is reported in Figure S4b. The OECT channel potential can be estimated by approximating the integral on the right-hand side of the Eq. S19 as a discrete sum of experimental swelling traces, obtained by varying the applied DC drain voltage across the channel. As an example, the swelling integral for the OECT working point defined by *V_G,DC_* = 0V and *V_D,DC_* = -0.2V can be approximated as $\int_{0}^{V_{D,DC}=-0.2 V} \bar{s}\left( \omega,x,V_{G,DC}=0 V,v_{D,DC} \right) dv_{D,DC}\approx\sum_{i} \bar{S_{i}}(\omega,x,V_{G,DC}=0 V,v_{D,DC})\cdot\Delta v_{D,DC}$ where $\bar{S_{i}}\left( \omega,x,V_{G,DC}=0 V,v_{D,DC} \right)$ are the strain profiles reported in Figure S4d with $v_{D,DC}$ ranging from -0.05 to -0.2 V with step $\Delta v_{D,DC}$ = -0.05 V.

Channel potential profiles obtained at different *V_G,DC_* are reported in Figure S4c. Data traces overlap in correspondence to the drain electrode ad assume a value coincident with the applied DC drain voltage (*V_D,DC_* = -0.2 V). Consistently with strainline-scan presented in Figure S4b, data show a strong dependence on the OECT working point. When the OMIEC channel is highly doped, the channel potential linearly drops along the PEDOT:PSS film, but increasing the gate voltage causes a rapid decrease of the channel potential and a consequent reduction of the local PEDOT:PSS swelling. Eq. S19 can also be used to study the evolution of the channel potential at different *V_D,DC_*. Voltage profiles reported in Figure S4e assume a minimum value in correspondence to the source electrode and increase along the channel until reaching the applied DC drain potential. The slight deviation from linear behavior observed at larger drain voltages (*V_D,DC_* = -0.2 V) inidcates that the OECT operation approaches the saturation regime.

**T6. Electrochemical impedance spectroscopy of OMIEC channels**

We used electrochemical impedance spectroscopy to measure the volumetric capacitance of BBL and PEDOT:PSS channels. Measurements were performed by shorting the source and the drain electrodes and applying an AC voltage (with amplitude 100 mV and frequency *f*) to the OMIEC layer with respect to the Ag/AgCl gate. The resulting impedance spectra (Figure S2) were fitted using the equivalent circuit shown in Figure S2a, which consists of a transmission line representing the OMIEC channel (as depicted in Figure 1a) with impedance ^[11]^

$$Z_{TLM}=\sqrt{r_{el}z_{0}} coth\left( \frac{r_{el}}{z_{0}} \right) (S24)$$

where *r_el_* and *z_0_* are defined as in Eq. S16. The transmission line circuit was placed in parallel with an RC circuit modeling charge accumulation in the regions of the mixed conducting film overlapping the source and drain electrodes. The volumetric capacitance *c_v_* of the mixed conductors was set as a constant for the whole film. Impedance fitting provides *c_v_* = 720 ± 40 Fcm^-3^ for BBL and *c_v_* = 40 ± 5 Fcm^-3^ for PEDOT:PSS respectively, consistently with literature values.^[12,13]^

**T7: Analysis of DC OECT characteristics**

The threshold voltage of PEDOT:PSS (Figure S3a) and BBL (Figure S3b) OECTs were measured from experimental DC transistor curves. In linear regime, the OECT behavior can be locally modeled using the Bernards-Malliaras model^[14]^

$$I_{D,DC}=\frac{W}{L}t_{h}c_{v}\mu_{el}\left[ \pm(V_{G,DC}-V_{t}) \right]V_{D,DC} (S25)$$

where the “-” sign refers to p-type devices operating in depletion mode and the “+” sign refers to n-type devices operating in accumulation mode. We obtain *V_t_* = (0.60±0.12) V for PEDOT:PSS and *V_t_* = (0.11±0.08) V for BBL OECTs, respectively. The electronic resistivity of OECT devices was finally calculated as

$$\rho_{el}= \frac{V_{D,DC}Wt_{h}}{I_{D,DC}L} (S26)$$

where *I_D,DC_* is the drain current and *V_D,DC_* the applied drain voltage. The average electronic mobility is given by

$$\left\langle\mu_{n} \right\rangle= \frac{1}{ne\rho_{el}} (S27)$$

where $n=\frac{c_{v}}{e}\left( V_{G,DC}-V_{t} \right)$ is the carrier density in the film.

**Bibliography**

[1] F. Bonafè, F. Decataldo, T. Cramer, B. Fraboni, *Advanced Science* **2024**, *11*, 2308746.

[2] R. Giridharagopal, L. Q. Flagg, J. S. Harrison, M. E. Ziffer, J. Onorato, C. K. Luscombe, D. S. Ginger, *Nature Mater* **2017**, *16*, 737.

[3] F. Mariani, F. Conzuelo, T. Cramer, I. Gualandi, L. Possanzini, M. Tessarolo, B. Fraboni, W. Schuhmann, E. Scavetta, *Small* **2019**, *15*, 1902534.

[4] S. Tanwar, R. Millan-Solsona, S. Ruiz-Molina, M. Mas-Torrent, A. Kyndiah, G. Gomila, *Advanced Materials* **2024**, *36*, 2309767.

[5] A. Kyndiah, M. Checa, F. Leonardi, R. Millan-Solsona, M. Di Muzio, S. Tanwar, L. Fumagalli, M. Mas-Torrent, G. Gomila, *Advanced Functional Materials* **2021**, *31*, 2008032.

[6] F. Bonafè, M. Bazzani, B. Fraboni, T. Cramer, *Nature Communications* **2025**, *16*, 2499.

[7] W. H. Hayt, *Engineering Electromagnetics*, New York : McGraw-Hill, **1989**.

[8] F. Bonafè, C. Dong, G. G. Malliaras, T. Cramer, B. Fraboni, *ACS Appl. Mater. Interfaces* **2024**, *16*, 36727.

[9] B. D. Paulsen, S. Fabiano, J. Rivnay, *Annual Review of Materials Research* **2021**, *51*, 73.

[10] C. Zhang, L. Margotti, F. Decataldo, A. Piccioni, H. Wang, B. Fraboni, Y. Li, T. Cramer, *Advanced Electronic Materials* **2024**, *10*, 2300762.

[11] R. de Levie, *Electrochimica Acta* **1963**, *8*, 751.

[12] C. Zhang, J. Meng, Y. Chen, C. Hou, K. Li, Q. Zhang, Y. Li, H. Wang, *J Mater Sci* **2023**, *58*, 15727.

[13] F. Bonafè, F. Decataldo, I. Zironi, D. Remondini, T. Cramer, B. Fraboni, *Nat Commun* **2022**, *13*, 1.

[14] D. A. Bernards, G. G. Malliaras, *Advanced Functional Materials* **2007**, DOI 10.1002/adfm.200601239.
